# Perovskite Nanoparticles as an Electrochemical Sensing Platform for Detection of Warfarin

**DOI:** 10.3390/bios12020092

**Published:** 2022-02-03

**Authors:** Anees Ahmad Ansari, Manawwer Alam

**Affiliations:** 1 King Abdullah Institute for Nanotechnology, King Saud University, Riyadh 11451, Saudi Arabia; 2 Department of Chemistry, College of Sciences, King Saud University, Riyadh 11451, Saudi Arabia; maalam@ksu.edu.sa

**Keywords:** perovskite, electro-catalysts, crystal structure, sensing, warfarin

## Abstract

Chemically prepared PrAlO_3_ perovskite nanoparticles (NPs) were applied for the electrochemical detection of warfarin, which is commonly utilized for preventing blood clots, such as in deep vein thrombosis. PrAlO_3_ perovskite NPs were synthesized by the co-precipitation process at environmental conditions. Crystallographic structure, phase purity, morphological structure, thermal stability, optical properties, and electrochemical characteristics were investigated by X-ray diffraction (XRD), transmission electron microscopy (TEM), thermogravimetric analysis (TGA), Fourier transform infrared (FTIR) spectroscopy, UV-visible analysis, and cyclic voltammetry techniques. TEM micrographs showed the highly crystalline structure, smooth surface, irregular shape, and size of nanocrystalline particles with an average size of 20–30 nm. Particularly crystalline perovskite NPs were pasted on glassy carbon electrodes (GCE) to electrochemically detect the warfarin contents in liquid samples. The fabricated electrode was electrochemically characterized by different parameters such as different potential, scan rates, same potential with seven consecutive cycles, time response, real-time sample analysis, and as a function of warfarin concentration in phosphate buffer solution (0.1 M PBS, pH 7.2). The electrochemical electrode was further verified with various potentials of 5, 10, 20, 50, 100, and 150 mV/s, which exhibited sequential enhancements in the potential range. For detecting warfarin over a wide concentration range (19.5 µM–5000 µM), the detection devices offered good sensitivity and a low limit of detection (19.5 µM). The time-dependent influence was examined using chronoamperometry (perovskite NPs/GCE) in the absence and presence of warfarin at four distinct voltages of +0.05 to +1.2 V from 0 to 1000 s. The repeatability and reliability of the constructed electrochemical sensing electrode were also evaluated in terms of cyclic response for 30 days, demonstrating that it is substantially more reliable for a longer period. The fabricated perovskite NPs/GCE electrodes could be employed for the rapid identification of other drugs.

## 1. Introduction

Warfarin is a commonly used medicine to prevent the clots in blood in individuals with abnormal heartbeats, and therefore it is called anti-coagulant or blood thinner medicine [1]. It improves blood circulation throughout the arteries. This implies that the bloodstream becomes minimized and prone to form potentially fatal clotting factors. On the basis of its strength, warfarin is commercially presented in different colors, although the size and shape of the tablets may vary in terms of manufacturing companies. However, warfarin has some side effects such as unusual headache, pain in joints, swelling, unusual bleeding from hemorrhoids, reddish or rust-colored urine, dizziness, difficulty in breathing, and uncontrolled bleeding from wounds and cuts, which could create some other heart problems, and therefore there is the need to examine warfarin contents in the human body. Because of human issues and health warnings, it is necessary to develop sensitive, cheap, fast, and accurate detection methods to monitor the drug contents in blood samples. Various techniques such as liquid chromatography, thin-layer chromatography, and mass chromatography have been developed, but lack of accuracy, detection limit, and sensitivity of the instruments limit their use in the detection of the drug contents [2,3,4,5]. Electrochemical techniques have become increasingly popular in recent years because of their low cost, quick reaction, ease of operation, high accuracy results, portability, and ability to detect very low concentrations in liquids. 

Currently, oxygen-derived nanomaterials are acquiring an enormous interest in the field of applied material as well as technological fields, owing to their superior optical and electrical, magnetic, and catalytic capabilities [6,7,8,9,10,11,12,13,14,15]. Perovskites (ABO_3_) are oxygen-deficient compounds, in which A is a larger size connected with 12 coordination sites and B has a six folded coordination and reduced cation in diameter with O_2_ anion [16,17]. The bond stretching limited co-doping of the A-site by transition metal ions with dissimilar valence causes a structural defect and changes the valence of the B-site to fulfill the chemical charge balance of the perovskite structure [18,19,20]. It is the primary source of the ABO_3_-derived oxide’s exceptional catalytic oxidation achievement. In the literature, different metal combinations have been developed for the preparation of perovskite structures for specific applications [18,19,20,21,22,23,24,25,26,27]. Additionally, perovskites have been successfully employed in various technological applications, such as catalysts [18,19,20], photocatalysts [28,29], water splitting [30,31,32], supercapacitor [33,34,35], chemical reductions/removal [36,37,38], solar cells [39,40,41], gas sensing [42,43,44], and chemical sensing device development [45,46,47,48,49,50]. Karuppiah et al. fabricated graphene nanosheet-wrapped mesoporous LaCeFeMnO_3_ perovskite for lithium battery applications [51]. Zhang et al. designed LaFeCoO_3_-deposited SiO_2_ for the cleaning of wastewater [52]. Because of the high oxygen storage capacity of perovskite lattices, they demonstrated excellent ionic mobility within the lattice, which enhanced the electronic and ionic conductivities of the materials. These ionic motilities within the lattice are highly exploitable in the fabrication of electro-catalytic, photo-catalytic, and electrochemical reaction-based technological applications. They demonstrated a wide range of applications in technological fields [18,19,20]. Furthermore, high-oxygen species mobility/conductivity improves the kinetics of oxygen electrode reactions, including oxygen reduction reaction and the oxygen evolution reaction in the fabrication of electrocatalytic-based devices. These novel features of the perovskite class of materials are highly recommended in the fabrication of electro-catalytic detection devices.

Hence, their use in the applied material sciences, wherein we successfully prepared PrAlO_3_ perovskite nanoparticles (NPs) by the co-precipitation process at environmental conditions. The crystal phase, crystallinity, and phase purity of the perovskite lattice were confirmed from the X-ray diffraction pattern (XRD). The morphology of the NPs was inspected by transmission electron microscope. The chemical composition, thermal stability, optical properties, surface characteristics, and electrochemical properties were defined from energy-dispersive X-ray (EDX) analysis, thermogravimetric analysis (TGA), UV-visible absorption spectrum, Fourier transform infrared (FTIR) spectrum, cyclic voltammetry (CV), and electrochemical impedance spectroscopic (EIS) techniques. Additionally, as-synthesized perovskite NPs were assembled on a glassy carbon electrode (GCE), and a three-electrode system was used for the electro-catalytic detection of warfarin medicine in solution media. The fabricated perovskite NPs/GCE electrode was used for the investigation of various electro-catalytic parameters such as the impact of concentration, the influence of potential, scan rate, real sample analysis, stability, reproducibility, chronoamperometry analysis, and electrochemical impedance analysis for the sensitive electro-catalytic detection of warfarin in phosphate buffer solution (PBS). 

## 2. Experimental

### 2.1. Materials

Praseodymium oxide (Pr_6_O_11_, 98.9% BDH Chemicals, Poole, UK) and aluminum nitrate (99.9% E-Merck, Darmstadt, Germany) were procured for synthesis purposes. Pr_6_O_11_ was converted into metal nitrate by dissolving in dilute nitric acid. The citric acid (E-Merck, Germany) and NH_4_OH (BDH Chemicals, England) were used directly without any further purification. Milli-Q H_2_O (Millipore, Burlington, MA, USA) was used for the synthesis and characterization of the sample.

### 2.2. Synthesis of PrAlO_3_ Perovskite NPs

Briefly, for the synthesis of PrAlO_3_ perovskite NPs, an equal volume, 6 mg of the Pr(NO_3_)_3_6H_2_O and 5.172 mg of Al(NO_3_)_3_xH_2_O, were dissolved separately in 50 mL Milli-Q water. Separately, an equal volume of citric acid dissolved in Milli-Q H_2_O was introduced into the magnetically stirred hot solution, and the solution mixture was kept on a hot plate for constant stirring at 80 °C for 30 min. After homogeneous mixing of citric acid with praseodymium nitrate, separately prepared Al(NO_3_)_3_xH_2_O solution was mixed into the ongoing mechanically stirred hot reaction mixture. This reaction mixture was kept on a hot plate for 2 h with mechanical stirring. In this stirring hot reaction mixture, ammonium hydroxide solution was introduced slowly for precipitation. An occurred precipitate was separated by centrifugation, washed with Milli-Q water to remove unreacted reactants, and dried in the oven overnight at 80 °C. For complete conversion of metal oxide into perovskite material, the occurred precipitate was annealed at 700 °C in the furnace for 5 h [18,20]. 

### 2.3. Characterization

X-ray diffraction (XRD) pattern was carried out by using a PANalytical X’PERT X-ray diffractometer with Cu *K*α radiation (λ = 1.54059 Å) at 50 kV and 200 mA at room temperature to monitor the phase purity and crystal phase of the as-prepared perovskite material. A field emission transmission electron microscope (FETEM, JEM-2100F, JEOL, Tozakishima, Japan) was utilized to inspect the morphological structure of the prepared product. Energy dispersive X-ray (EDX, JEM-2100F, JEOL, Tozakishima, Japan) analysis was carried out to assess the metal contents into the as-prepared perovskite product with an accelerating voltage of 200 kV. Thermogravimetric analysis was measured from a TGA/DTA, Mettler, Toledo AG instrument (Analytical CH-8603, Schwerzenbach, Switzerland). UV-visible absorption spectra were measured by a Perkin-Elmer Lambda-40 (Artisan technology group, Kansas, MO, USA) spectrophotometer. Fourier transform infrared spectrum was observed by a Perkin-Elmer spectrum 100 IR spectrometer. Cyclic voltammetry (CV) and electrochemical impedance spectroscopic (EIS) techniques were utilized to monitor the electro-catalytic behavior of the as-synthesized perovskite material. The electrochemical performance of the perovskite NPs was determined using a three-electrode cyclic voltammogram, Autolab Potentiostat/galvanostat PGSTAT204(Metrohm AG Ionenstrasse CH-9100, Herisau, Switzerland) Metrohm Autolab analyzer (PGSTA30, Metrohm Autolab, Utrecht, The Netherlands). The working electrode was changed from a PrAlO_3_/glassy carbon electrode (GCE), platinum (Pt) was utilized as a counter electrode, and Ag/AgCl was employed as a reference electrode in this three-electrode arrangement. The working electrode was made by combining a small amount of perovskite NPs with insulation material nature butyl carbitol acetate at an 80–20 molar ratio, then depositing the slurry onto the GCE and drying it at 60 °C for 30–45 min to achieve an unchangeable protective layer over the perfect surface of the electrode with an effective surface area of 0.07 cm^2^. All of these electrochemical studies were carried out in natural settings.

## 3. Results and Discussion

### 3.1. Crystallographic and Morphological Structure

**Figure 1:**
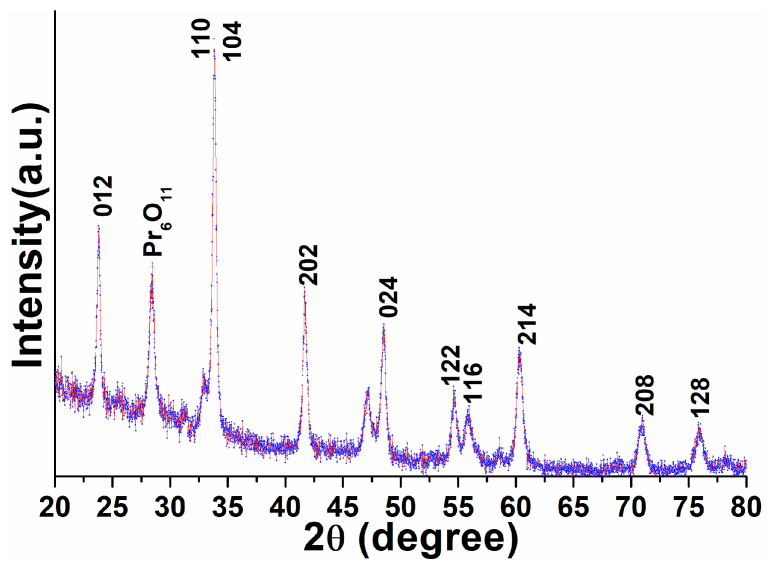
X-ray diffraction pattern of the as-prepared PrAlO_3_ perovskite NPs.

A typical X-ray diffraction pattern of the powder sample was utilized to examine the phase purity, crystal structure, and crystallinity of the as-prepared perovskite NPs. The XRD pattern shown in Figure 1 exhibited all reflection planes assigned to the (012), (110), (104), (202), (024), (122), (116), (214), (208), and (128), which corresponded to the pure hexagonal phase of the perovskite structure (JCPDS card no. 032-0484) [18,20,53]. The XRD finding indicated no reflection line for Al species, which could have been due to homogenous distribution within the perovskite lattice. It denotes the development of uniform Pr-Al-O solid form. Noticeably, the width of the XRD reflection planes was broadened, which indicated the dimensions of the particles were in nano-scale form with crystallinity. Through using XRD analysis, we determined the lattice constants of the resulting NPs to be 0.5280 Å, 0.5379 Å, and 0.52998 Å, being significantly lesser than their bulk NPs (5.411 nm). The average crystalline size of the perovskite NPs was estimated from the Debye–Scherrer equation by using the half-width full maxima of the most dominant diffraction peak located at 2*θ* = 33.73° to be 28 nm [19,20]. 

**Figure 2:**
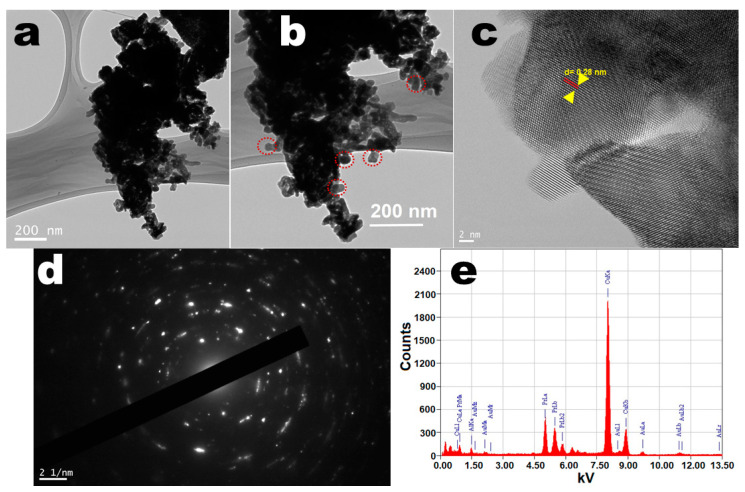
TEM micrographs of the (**a**,**b**) low-resolution pictures; (**c**,**d**) selected area electron diffraction pattern and high-resolution TEM pictures with lattice spacing; (**e**) EDX analysis of the PrAlO_3_ perovskite NPs.

The morphological structure of the perovskite NPs was determined through a field-emission transmission electron microscope (FE-TEM) to observe the dimension and shape of the as-prepared particles. The TEM images shown in Figure 2a,b demonstrated well-resolved highly crystalline size, rough surface, non-spherical shape, and narrow distributed sizes ranging from 20 to 30 nm, which were closely matched with the observed XRD results. The smooth surface with irregular shape and size and high aggregation of the crystals were possibly due to calcination at high temperature. In the high-resolution TEM picture, the crystal lattice fringes were visible, suggesting the formation of single-crystal high crystallinity in perovskite NPs (Figure 2c,d). The lattice fringes on the single perovskite NPs can be readily seen in the high-resolution TEM picture, suggesting that the produced NPs were extremely crystalline. The width within lattice fringes was observed to be 0.28 nm, which matched the d-spacing for the hexagonal perovskite phase (110) lattice planes [18,19,20,54]. The selected area electron diffraction pattern illustrated the dotted polycrystalline reflection rings assigned to the hexagonal phase of the PrAlO_3_ crystal structure, which were in accordance with the XRD observations (Figure 2d). Energy-dispersive X-ray (EDX) analysis was applied to monitor the phase purity and the existence of the chemical constituent in the perovskite NPs lattice. The EDX spectrum shown in Figure 2e illustrated the existence of all elements corresponding to Pr, Al, and O in the PrAlO_3_ crystal lattice. According to the EDX absorption lines, the metal ions were uniformly spread within the crystal lattice. Two highly prominent peaks were observed for C and Cu, belonging to the carbon-coated Cu grid on which the sample was deposited for scanning. There was no other contaminant peak visible in the EDX spectrum, implying phase purity and consequently the synthesis of single-phase UCNPs. These findings are also in line with the XRD, FTIR, and TGA findings.

### 3.2. Thermal Analysis

**Figure 3:**
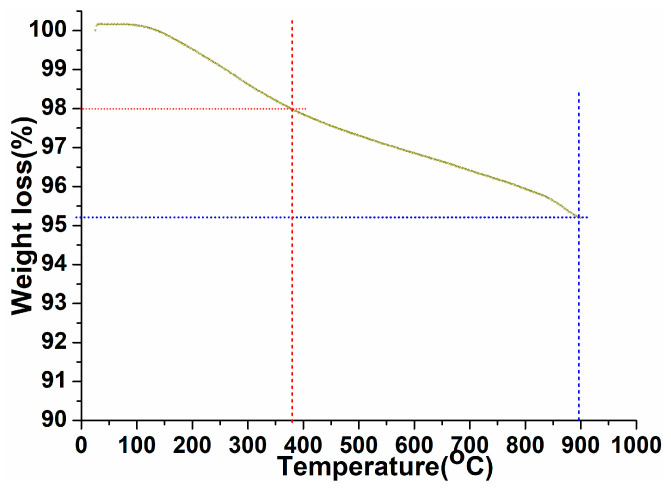
Thermogravimetric analysis of the as-synthesized PrAlO_3_ perovskite NPs.

A thermogravimetric study was carried out to examine the thermal stability, phase purity, and surface-fastened organic moieties over the exterior of the as-prepared perovskite NPs under ambient to 900 °C temperature in an N_2_ atmosphere with a heating rate of 10 °C/min (Figure 3). As shown in Figure 3, the thermogram revealed first ~2% weight loss in between 25 and 380 °C, assigned to the surface attached organic moieties over the perovskite crystal lattice [55,56]. As exhibited in the thermogram after removal of the organic moieties, the curve displayed progressive weight loss up to 900 °C with weight loss of ~2.7%. This indicated the elimination of crystalline and non-crystalline water molecules and some tangling bonds in the perovskite crystal lattice, which were attached within the crystal lattice in different covalent or ionic bonding forms [18,20]. These observed results are in accordance with the XRD pattern.

### 3.3. Optical Properties

**Figure 4:**
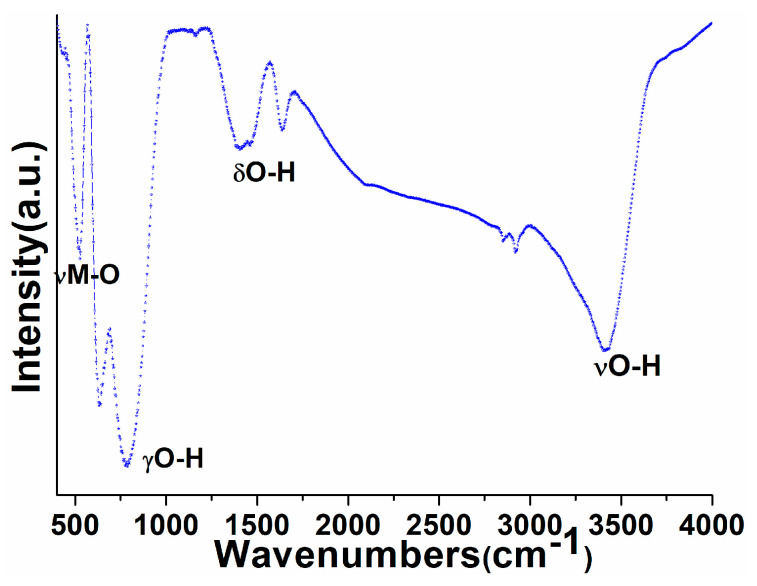
FTIR spectrum of the as-synthesized PrAlO_3_ perovskite NPs.

FTIR spectrum was recorded to investigate the phase purity and surface-attached organic or water impurities. The infrared spectrum of perovskite NPs demonstrated broadband in between 3025 and 3703 cm^−1^, which corroborated the asymmetrical/symmetrical stretching vibrational mode of the surface-anchored water molecules (Figure 4). Additionally, two infrared absorption peaks located at 1630–1650 and 785 cm^−1^ corresponded to the δOH (bending) and γOH (scissoring) vibrational modes of the physically surface-adsorbed un-dissociated form of water moieties, respectively [57,58,59]. The νM-O expanding vibrational mode, which validated the development of metal oxide frameworks, was assigned to the detected infrared absorption peak at 519 cm^−1^ [60,61,62]. TGA findings corroborated the obtained FTIR spectrum explanations.

**Figure 5:**
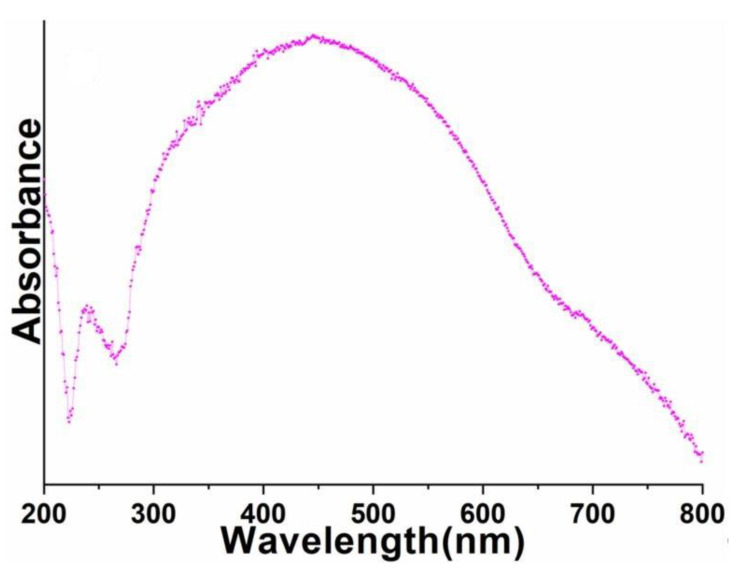
UV-visible absorption spectrum of the as-synthesized PrAlO_3_ perovskite NPs.

UV-visible spectrum was monitored to explore the optical properties of the as-prepared perovskite NPs. As shown in Figure 5, the absorption spectrum of the as-prepared perovskite NPs displayed a diffused absorption band between 300 and 800 nm. Notably, the well-recognized 4f-4f absorption transitions of Pr^3+^-ion were not detected in the UV-visible absorption spectrum of perovskite NPs (Figure 5). This could have due to the strong absorption of Al^3+^ ions in a similar visible region. 

### 3.4. Electrochemical Characterization

**Figure 6:**
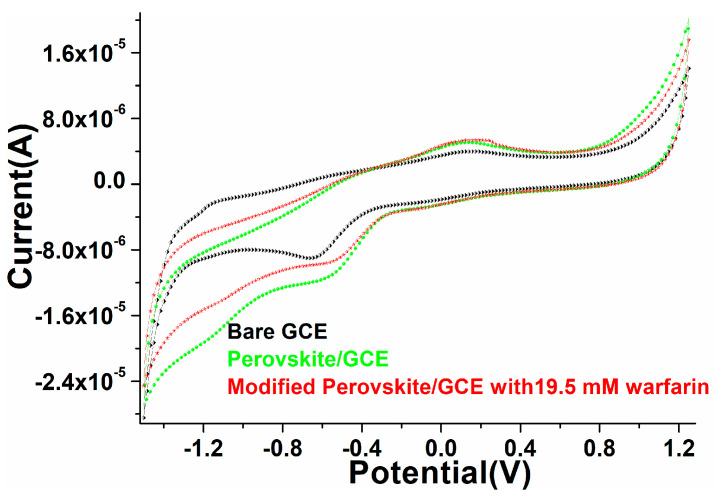
Electrochemical characterization through cyclic voltammogram of the as-constructed PrAlO_3_ perovskite NPs/GCE electrode.

Cyclic voltammetry was performed to explore the electrocatalytic properties of the as-prepared perovskite matrix. As discussed in the fabrication of the film/electrode in the experimental section, a layer of the perovskite NPs was coated (area 3 × 3 cm^2^) over the GCE to analyze the electrochemical performance of the deposited film/electrode, as shown in Figure 6. When we evaluated the blank electrode (scan rate 100 mV/s) in phosphate buffer solution (PBS; 0.1M, pH 7.2), a slight hump in the redox curve was observed. Similarly, after coating the perovskite NPs layer over the electrode, we observed a significant change in the redox reaction (curve), which indicated that a substantial change in the oxidation reduction hump validated the increased reactivity of the fabricated electrode. Similarly deposited electrode was scanned in the presence of warfarin in PBS, the oxidation and reduction curve exhibited a prominent change, which was assigned to the enhanced sensing activity of the fabricated electrode (Figure 6). An alteration in the anodic and cathodic peak current in the presence of warfarin suggested that the fabricated electrode facilitated the communication of electrons between the electrode surface and the electro-catalytic analyte [17,63].

**Figure 7:**
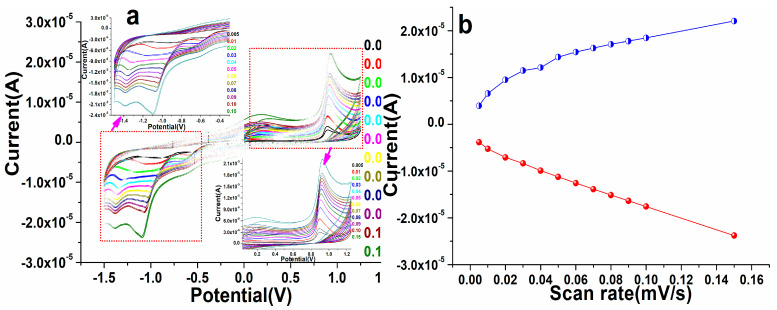
(**a**,**b**) Cyclic voltammogram of the as-fabricated PrAlO_3_ perovskite NPs/GCE electrode as a function of different scan rate in ascending order.

To examine the redox potentiality of the as-fabricated perovskite NPs, we tested the electrode with different potential ranges from 5, 10, 20, 30, 40, 50, 60, 70, 80, 90, 100, and 150 mV/s in PBS at the environmental condition, as illustrated in Figure 7. As demonstrated in Figure 7, on increasing the potential/scan rate from 5 to 150 mV/s, we detected a significant change in the redox (anodic/cathodic) peak position of the modified perovskite NP electrode, showing that the modified electrode assisted in the transporting of the electron process. At the different potential of fixed concentrations of warfarin in 0.1 M PBS solution at pH 7.2, the peaks of potential shifted favorably at greater scan rates and negatively at lesser scan rates. This demonstrated the improvement in the current and potential of perovskite NPs electrodes for warfarin. A modest oxidation and reduction signal was noticed in the spectrum at first, indicating that there was insufficient potential, but when the parameters of potential changed, the increase in current was visible. This shift specified that the perovskite matrix was treated and that the NPs can significantly assist in the detection of warfarin in PBS solution. These observed findings showed that the electrostatic interaction between the as-fabricated perovskite NPs/GCE electrode and warfarin is kinetically regulated by diffusion [17,63,64,65]. The linear plots were also generated on the basis of the acquired results and their explanation connected to the different concentrations and potential to determine the ionization potential of the cathode (IPc) and anode (IPa) as well as the correlation coefficient (R^2^) [66,67]. The results showed that the R^2^ values for perovskite NPs/GCE were 0.998 and 0.989, respectively, in IPa and IPc (Figure 7).

### 3.5. Electrochemical Sensing Properties

**Figure 8:**
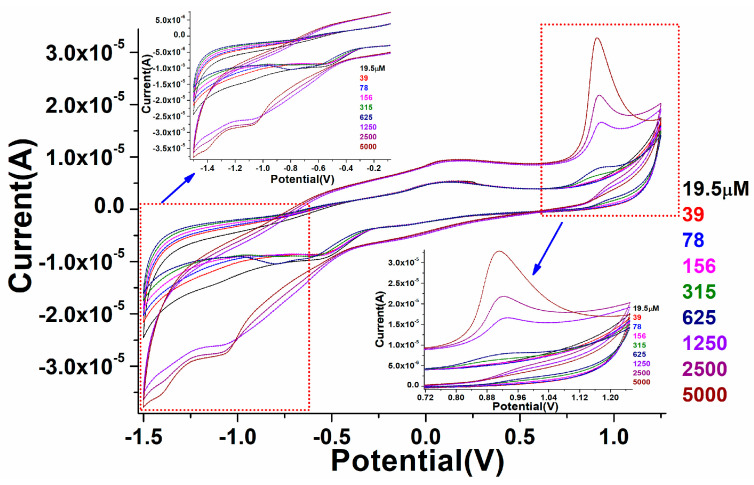
Cyclic voltammetry study of the as-fabricated PrAlO_3_ perovskite NPs/GCE as a function of warfarin concentrations in 0.1 M PBS solution.

The fabricated perovskite NPs/GCE was employed for the electro-catalytic detection of warfarin in 0.1 PBS solution. Perovskite/GCE electrode was scanned at 0.1 M, pH 7.2 PBS solution under constant 100 mV/s scan rate in the presence of different concentrations of warfarin, namely, 19.5, 39, 78,156, 315, 625, 1250, 2500, and 5000 µM. As displayed in Figure 8, on the addition of warfarin quantities in the potential range of −2.0–+2.0 V, a progressive difference in redox signals was noticed in the CV spectra. Furthermore, as the amount of warfarin increased, the linearity in the curve gradually increased in oxidation peaks. In increasing the concentration of warfarin, we found that the magnitude of the peak maxima was altered, which was due to the close relationship between the quantity of warfarin and the anodic/cathodic signals, as illustrated in Figure 8. It also means that when the amount of warfarin was added, the current generated by ion movement increased, simulating the quick transport of electrons to the conduction band. It was discovered that the perovskite NPs/GCE electrode efficiently catalyzed the electro-catalytic oxidation of warfarin because of the diffusion-controlled phenomenon. Figure 8a shows the calibration graph of warfarin concentration vs. peak current maxima, as well as the electro-catalytic detection parameters of the CV spectra that were experimentally observed.

As shown in Figure 8, a curve was generated to monitor the minimum and maximum concentration range in which fabricated perovskite electrodes worked efficiently. The fabricated electro-catalytic electrode showed the linearity curve with a detection range of 19.5 µM. According to this curve, the constructed electrode was sensitive to a warfarin concentration of 19.5 µM (LOD). When comparing a higher concertation scale to a lower one, we found the regression coefficient to be larger. 

**Figure 9:**
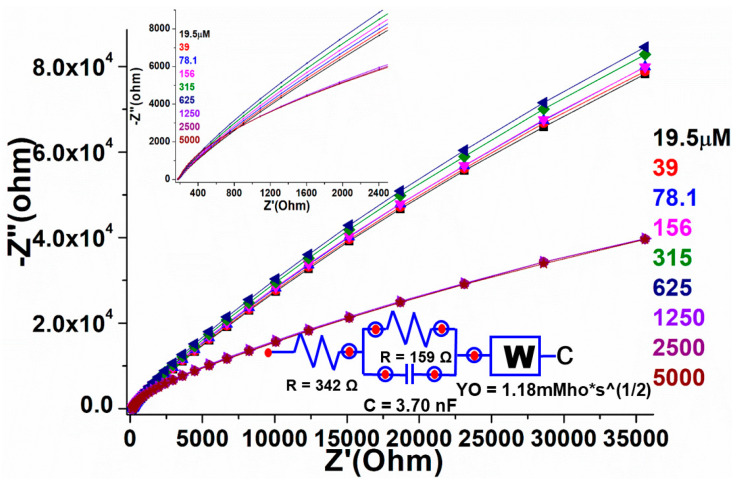
Electrochemical impedance spectroscopy of the fabricated PrAlO_3_ perovskite NPs/GCE as a function of warfarin concentrations in 0.1 M PBS solution.

A similar fabricated electrode was further characterized by the electrochemical impedance spectroscopic (EIS) method to validate the electrocatalytic properties of the as-designed film/electrode through resistance along with conductance of the analyte. In the Nyquist graph, the conductive characteristic is represented by the *x*-axis, while the resistive property is shown as the *y*-axis. According to earlier literature, the impedance addresses both high- and low-frequency zones. EIS technique was used to investigate the sensing performance of the manufactured electrode over a wide concentration range (19.5, 39, 78, 156, 315, 625, 1250, 2500, and 5000 µM in 0.1 M, pH 7.2 PBS) in the frequency ranges from 0.01 to 10 kHz (Figure 9). The charge transfer resistance, such as the obstacle posed by the electrode NPs to the communication of charge from the solution to the electrode that can be linked to the exterior change, is given by the Nyquist plot diameter. The straight-lined component of the curve is typical of lower-frequency spectra and reflected the process of diffusion-limited electron transfer, while the curve found at higher frequencies corresponded to the process of electron transfer rate. The curved spectra revealed a straight line in the blank sample, indicating that there was less frequency with the confined electron transfer method, but when the various concentrations of warfarin were used for the PBS and the EIS was analyzed, many curved semicircles were observed in the high-frequency region. This was similar to electron transport in a restricted process. The width of each semicircle showed the high frequency and resistance charge transfer (Rct), which was in charge of controlling the electron transfer rate at the electrode interface. It is a well-identified fact that the greater the semicircle curve, the higher the interfacial Rct, and thus the prepared/active NPs low electrical conductivity. The results in the form of curves showed that the diameter of the semicircle began to grow at low warfarin concentrations and grew in size as the concentration increased. This could have been due to high Rct (Ω) values in the perovskite/GCE electrode system. On the addition of warfarin doses, the charge transfer rate improved progressively, resulting in the straight line being gradually altered. These findings are consistent with the cyclic voltammetry sensing findings. We also used a circuit to compute the values of solution resistance (Rs (Ω)) and charge transfer resistance Rct (Ω). When the doses of warfarin in PBS were increased, the Rs(Ω) and Rct (Ω) values were steadily suppressed, indicating that the constructed detection device was good and reproducible. To show the difference in EIS plots with the increasing quantity of warfarin, we produced a plot using the calculated values of solution resistance (Rs (Ω)) and charge transfer resistance Rct(Ω) (inset in Figure 9).

### 3.6. Electrochemical Stability and Reproducibility

**Figure 10:**
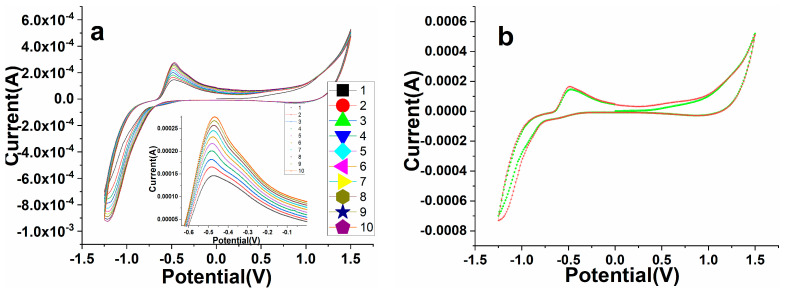
(**a**) Electrochemical reproducibility measured by CV at seven consecutive cycles of PrAlO_3_ perovskite NPs/GCE electrode in the presence of 19.5 μM (warfarin) in 0.1 M PBS at 100 mV/s. (**b**) Stability test first and after 30 days at the same conditions.

The repeatability and stability of the as-constructed perovskite NPs/GCE electrode were examined in terms of their cycle response, and the results are shown in Figure 10a,b. The tested data of perovskite NPs/GCE electrode in the existence of warfarin (19.5 µM) in 0.1 M PBS was observed at seven parallel repeatable cycles (Figure 10a) and stability (Figure 10b). It demonstrated that sequential measurements in the existence of warfarin had remarkable repeatability. From the first to the one week mark, the observation was evaluated for reproducibility (Figure 10a). Because the perovskite NPs/GCE electrode was retained in an ambient environment during this time, there was only a minor change in the form of the cycle response. The stability of the processed perovskite NPs/GCE electrode was also evaluated for a longer period (one month) (Figure 10b), confirming that the sensor is good and stable over a long time. The results revealed that the constructed electrode is very pleasing, repeatable, and long-lasting, making it suitable for testing industrial samples.

### 3.7. Impact of Time Response with Perovskite/GCE against Warfarin

**Figure 11:**
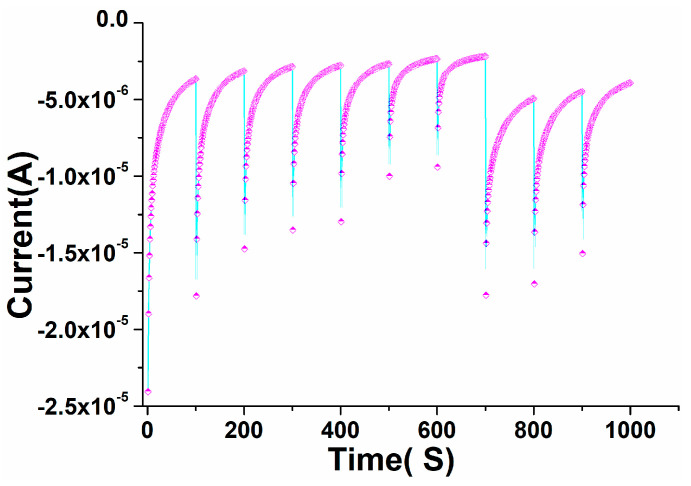
Chronoamperometric test for the PrAlO_3_ perovskite NPs/GCE electrode by subsequent additions of in warfarin in 0.1 M PBS.

The chronoamperometric curve was used to determine the electrocatalytic activity and consistency. To assess the detection selectivity and repeatability, we obtained a sequential temporal response for perovskite NPs/GCE in warfarin from 0 to 1000 s (Figure 11). The prepared perovskite NPs/GCE electrode was subjected to a systematic modification over time. At 100 s, 200 s, 300 s, 400 s, 500 s, 600 s, and 700 s, the current was gradually improved for the perovskite NPs/GCE electrode. The fabricated perovskite NPs/GCE was found to be specific, selective, and repeatable, as evidenced by the chronological and subsequent data. The developed perovskite NPs/GCE had enough durability and dependability for a long period, according to the time-dependent and specific investigation. The current–time phenomenon of the chronoamperometric graph showed that when the potential of the electrode increased, the current of the electrode decreased, indicating that the perovskite-based electro-catalyst was poisoned by intermediate species during the reduction process.

### 3.8. Real Sample Analysis

**Figure 12:**
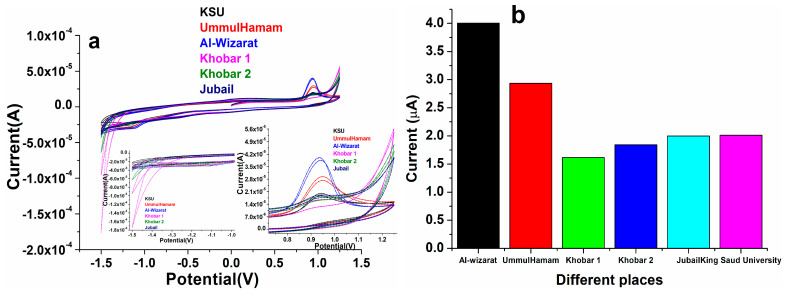
(**a,b**) Cyclic voltammetry spectra of the PrAlO_3_ perovskite NPs/GCE electrode upon injection of real samples and followed by continuous injections of 19.5 μM (warfarin) in 0.1 M PBS buffer (pH 7.2); (**b**) the data in graph form.

As displayed in Figure 12, the standard addition method was utilized to analyze the practical effectiveness of the as-designed electro-catalytic device in various H_2_O samples to determine its efficient adaptability in real samples. In real samples, the exact amount of warfarin was applied and evaluated using perovskite NPs/GCE at a constant potential. The recoveries varied from 98 to 103 percent at perovskite NPs/GCE electrode with the addition of warfarin standard solutions of various concentrations, representing that our constructed perovskite/GCE electrode is reliable for detecting warfarin in tap water(), as shown in Figure 12. 

## 4. Conclusions

In this article, we observed the electrochemical detection of warfarin drugs from the solution media through perovskite NPs/GCE as a platform. The chemically prepared perovskite was characterized by different spectroscopic techniques to verify its crystal phase, phase purity, surface chemistry, thermal stability, optical characteristics, and electrochemical properties. XRD, TGA, and FTIR spectral studies validated the formation of a single-phase, highly pure substance with excellent thermal stability. TEM micrographs confirmed the high-crystalline, smooth-surface nanocrystals with an average grain size of 20–30 nm. EIS and CV studies verified the electrochemical properties of the fabricated (perovskite NPs/GCE) electrode in PBS solution. The developed electrode was utilized to analyze the various concentrations (19.5 µM–5000 µM) of warfarin in PBS media. The chronoamperometry findings for developed modified perovskite NPs/GCE electrode in the available and non-available warfarin samples also supported the fact that the responses in terms of signal change were exhibited at specific voltages with defined time intervals (0–1000 s). According to the chronoamperometry findings, we found that the current rate was higher in the existence of warfarin in the solution than in the unavailable of warfarin in the PBS solution, indicating that fabricated perovskite NPs/GCE can work at a wide range of potentials. It was obvious from the experimental results that a distinct variation in cathodic and anodic signals was noticed, indicating that the constructed sensing electrode was accurate and helpful for bulk sample identification. The results on selectivity and repeatability (0–1000 s) indicated that the manufactured sensors had a large amount of durability for a long period of time. The real sample analysis was carried out on various water samples against as-fabricated perovskite NPs/GCE to validate the interfering results, which revealed that no substantial interference was observed, implying that the processed sensing electrode is highly responsive to ambient samples. The fabricated electrode may be appropriate for a variety of industrial tasks, such as determining the toxicity rate of dangerous drugs in concentrations ranging from low to high.

## Data Availability

Not applicable.
